# Delayed diagnosis of right-sided valve endocarditis causing recurrent pulmonary abscesses: a case report

**DOI:** 10.1186/s13256-019-2034-7

**Published:** 2019-04-19

**Authors:** Paul Bamford, Rajeev Soni, Levi Bassin, Anthony Kull

**Affiliations:** 1 Gosford Hospital, Holden Street, Gosford, NSW 2250 Australia; 2 University of Newcastle, Newcastle, NSW Australia; 3 Royal North Shore Hospital, St Leonards, NSW Australia

**Keywords:** Right-sided endocarditis, Pulmonary abscess, Pulmonary valve replacement

## Abstract

**Background:**

Pulmonary valve infective endocarditis is a rare diagnosis that is usually associated with immunocompromised states or structurally abnormal hearts. It is unusual for it to occur in structurally normal hearts or to cause recurrent symptoms after targeted antibiotics. Although guidelines suggest follow-up with repeat echocardiography and inflammatory marker surveillance, this case demonstrates that these are not always useful investigations, and instead imaging of the chest may be more appropriate.

**Case presentation:**

We describe a case of a 74-year-old man who presented with respiratory symptoms and was originally misdiagnosed with pneumonia but later found to have a large pulmonary valve vegetation caused by *Streptococcus mitis*. Despite initially responding to antibiotic therapy, the vegetation continued to cause pulmonary emboli and cavitating lung abscesses months later, necessitating pulmonary valve replacement.

**Conclusions:**

This case demonstrates that pulmonary valve endocarditis can present atypically with recurrent respiratory symptoms, and in such cases, echocardiography should be considered to investigate for right-sided infective endocarditis. In addition, despite correct treatment, with normalization of inflammatory markers and improvement in vegetation size, infective endocarditis can continue to cause systemic symptoms. Finally, clinicians should consider chest computed tomography routinely as part of right-sided infective endocarditis follow-up.

## Introduction

Pulmonary valve (PV) infective endocarditis (IE) accounts for 1.5–2% of IE cases, occurring ten times less frequently than tricuspid valve endocarditis [1, 2]. It is postulated that right-sided IE is less common, owing to fewer valvular abnormalities. The left side of the heart has higher hemodynamic pressures and more congenital abnormalities that result in greater endothelial disruption with increased platelet and fibrin deposition, serving as a nidus for pathogen adhesion. It is unclear what predisposes completely normal valves to IE aside from pathogens’ virulence factors that are thought to play a role in establishing infection [3–5]. Most cases of PV IE have associated tricuspid valve IE [6]. Although often associated with immunocompromised states, including intravenous drug use or structurally abnormal hearts, in 28% no risk factor is identified [4]. In such cases, *Staphylococcus aureus* and streptococcus viridans have been found to be the most likely pathogens [7, 8].

In this paper, we report a case of a 74-year-old man originally misdiagnosed with multilobar pneumonia who was eventually found to have a large PV vegetation as the source. This case is unusual in that despite antibiotics targeted to a susceptible *Streptococcus* sp., he had recurrent septic emboli and a cavitating lung abscess, which necessitated PV replacement. This case demonstrates the importance of performing cardiac imaging in patients with recurrent respiratory symptoms, and it highlights the need to perform imaging of the chest during follow-up to monitor for systemic complications.

## Case presentation

A 74-year-old male retired accountant with a background of asthma, atrial fibrillation, and gout presented to our emergency department with syncope following an insidious 6-month history of systemic symptoms. He had had intermittent fevers, 15-kg weight loss, general malaise, regular diaphoresis that occurred day and night, nausea, vomiting, diarrhea, and a nonproductive cough with sporadic morning hemoptysis. His exercise tolerance had reduced from unlimited walking capacity to breathlessness after roughly 2 km. His medications included rivaroxaban, verapamil, digoxin, and fosinopril. He had received a short course of prednisolone 25 mg daily for a flare of gout 3 weeks prior. He had a 50-pack-year ex-smoking history, having given up smoking 30 years prior. He lived independently with his wife. He had undergone outpatient chest computed tomography (CT) 2 months earlier that showed consolidation in the left lower lobe and a peripheral opacity in the right lung base measuring 14 mm by 12 mm. He had received several courses of oral antibiotics, including amoxicillin for 10 days and doxycycline for 2 weeks for presumed pneumonia. Because of his ongoing cough, he had a repeat CT scan 1 month later that showed resolution of the consolidation but no change in the peripheral opacity. His general practitioner had then referred him to a respiratory specialist, who felt that his illness was in keeping with a pneumonia that was now resolving. He advised withholding fosinopril, cessation of antibiotics, repeat CT scan in 3 months, and follow-up in 3 weeks. Prior to this appointment, he had had the syncopal episode that led to this presentation.

**Fig. 1:**
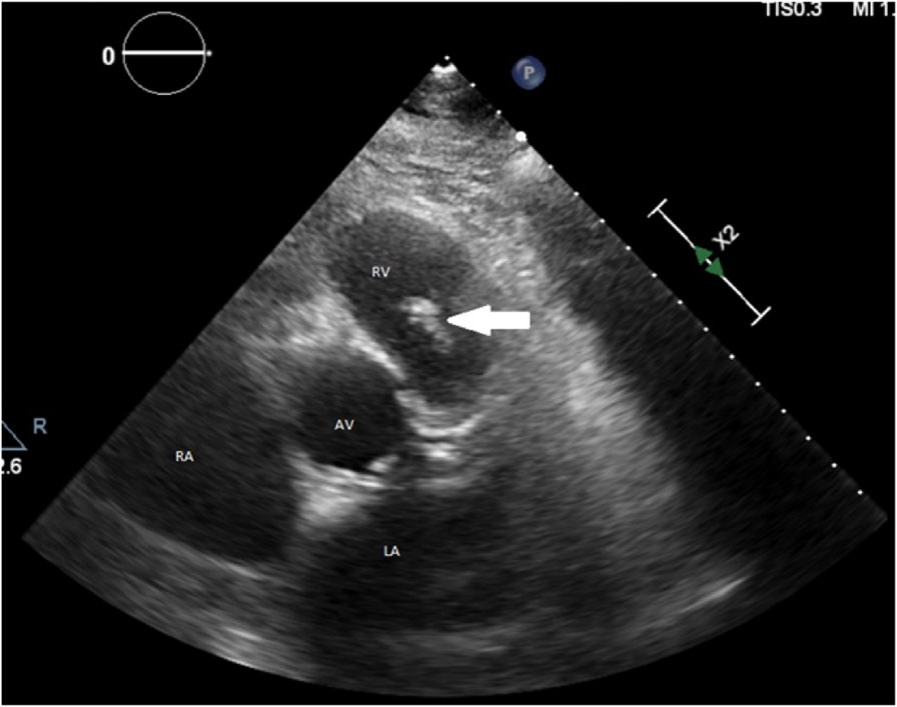
Transthoracic echocardiography parasternal short-axis view revealing a large pulmonary valve mass (*arrow*). *AV* Aortic valve, *LA* Left atrium, *PA* Pulmonary artery, *RA* Right atrium, *RV* Right ventricle

**Fig. 2:**
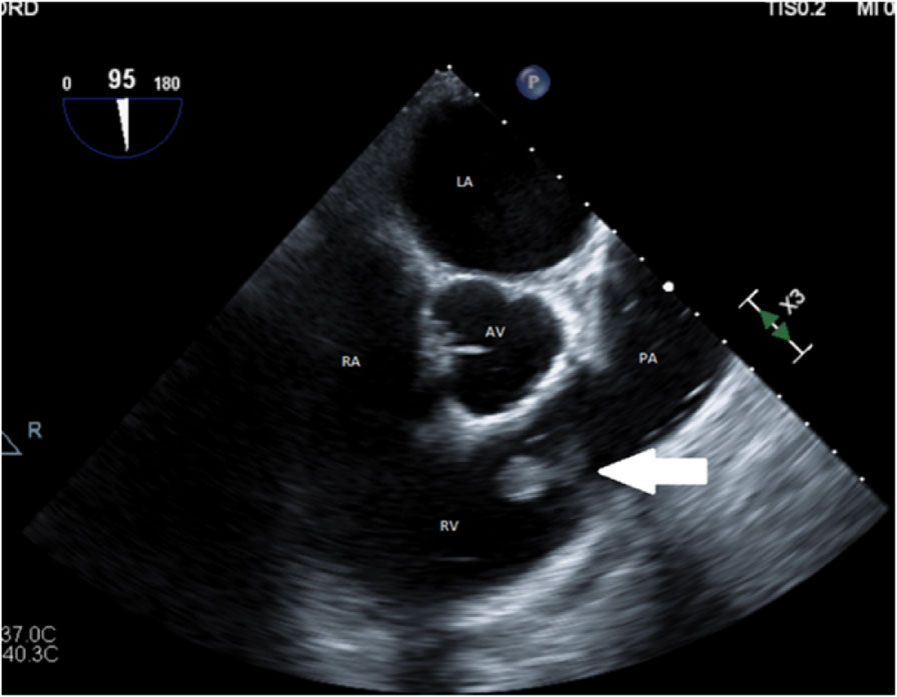
Transesophageal echocardiography showing vegetation (*arrow*) between right ventricle (RV) and pulmonary artery (PA). *AV* Aortic valve LA Left atrium, RA Right atrium

On arrival to the emergency department, he felt washed out, with vital signs that were notable for low-grade fever of 38.3 °C, sinus tachycardia to 130 beats per minute, and fluid-responsive hypotension (82/45 mmHg), and his physical examination was largely unremarkable. Investigations revealed a white blood cell (WBC) count of 10.6 × 10^9^/L (neutrophil count 9.1 × 10^9^/L, lymphocyte count 0.9 × 10^9^/L), C-reactive protein (CRP) 119 mg/L (normal range 0–5 mg/L), *Streptococcus mitis* on blood cultures (penicillin minimum inhibitory concentration [MIC] 0.030 mg/L [susceptible = MIC < 0.5 mg/L]), and transthoracic echocardiography (TTE) and transesophageal echocardiography (TEE) demonstrating a mobile 30 × 25-mm vegetation on the PV that extended into the right ventricular outflow tract (Figs. 1 and 2). Complete destruction of the anterior cusp with severe pulmonary regurgitation was seen. Initial management included intravenous benzylpenicillin and gentamicin for a total of 4 and 2 weeks, respectively. A surgical opinion was obtained, but a trial of medical management was advised. The patient felt better, and his inflammatory markers normalized.

**Fig. 3:**
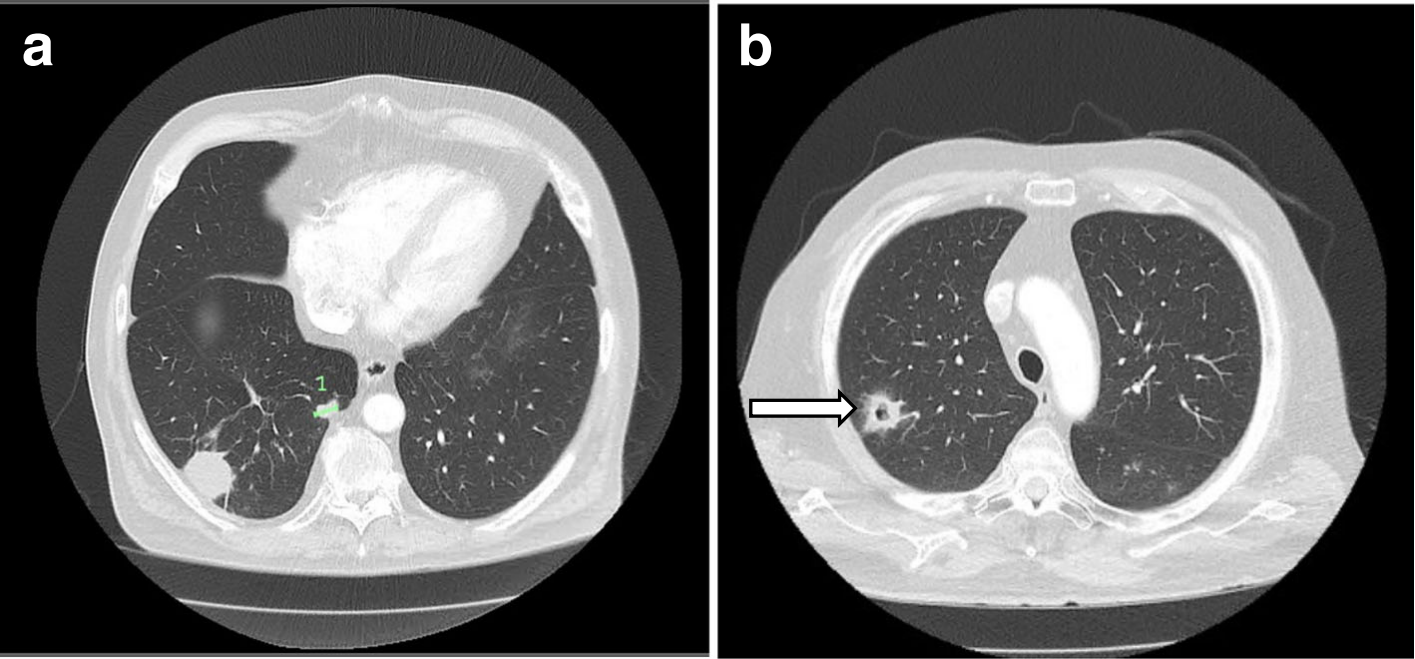
Repeat computed tomographic scans of the chest showing multiple emboli and abscesses despite antibiotics. **a** A 37-mm opacity in the right lower lobe posteriorly and inferiorly, abutting the pleura and an adjacent 14-mm opacity (*highlighted 1*). **b** An upper lobe abscess (*arrow*)

Two months after antibiotic cessation, the patient still felt well, with a normal WBC count and CRP of 5.3 mg/L (normal range 0–5 mg/L). Cardiac and chest imaging was performed to check resolution of lesions. TTE showed a reduction in the PV vegetation to 29 × 9 mm. A follow-up chest CT scan, however, showed new nodular lesions thought to be due to embolization (Fig. 3a), suggesting persistent endocarditis. Repeat blood culture results remained negative.

After 3 months, he again began to feel lethargic with breathlessness on exertion. His examination was unremarkable, but his WBC count was 16.1 × 10^9^/L and CRP was 182 mg/L. Results of five sets of blood cultures were negative, and the vegetation size had improved on TTE to 23 × 10 mm. He was then started on a 2-week course of amoxicillin and clavulanic acid. Again he began to feel well, with improvement in his CRP and WBC count, so he was discharged to home.

Repeat outpatient CT of the chest 1 month later showed new lesions, including a cavitating lesion (Fig. 3b). In light of the recurrent lung lesions, he underwent surgical PV replacement. His PV had been destroyed with a perforated anterior leaflet that was completely encompassed by a large vegetation. The other leaflets were untouched. After valve and vegetation excision, a 27-mm Carpentier-Edwards PERIMOUNT tissue valve (Edwards Lifesciences, Irvine, CA, USA) was inserted. Histology confirmed IE with expansile inflammatory masses composed of fibrin and neutrophils. Small clusters of degenerate bacterial cocci were noted, but a valve culture revealed no growth. The patient made a good recovery on intravenous benzylpenicillin and oral clindamycin. TTE showed a normal-functioning bioprosthetic PV. Over the following weeks, the patient’s radiographic and inflammatory markers normalized.

## Discussion

PV IE diagnosis is based on clinical findings that include fevers, pulmonary regurgitation, positive blood cultures, and echocardiographic features of pulmonary vegetation. Systemic embolization is more common with right-sided IE. In a large prospective study, the majority (53%) of patients with right-sided IE had systemic emboli at presentation, compared with 34% of those with mitral and aortic valve IE [9]. Because a majority of these patients present with respiratory symptoms, unless there is a high index of suspicion, the diagnosis may be delayed (as in our patient). An echocardiogram should be considered in this clinical scenario. Sensitivity of TTE has been estimated at 30–63% with specificity of 91–100%, and TEE has 87–100% sensitivity with 91–100% specificity [10].

Our patient was unusual in that despite responding to antibiotic therapy with multiple negative blood cultures and normalization of his CRP, he had new septic emboli months after treatment. European Society of Cardiology 2015 guidelines, although recommending surveillance echocardiography and monitoring of inflammatory markers for infection relapse, do not mention chest imaging as part of follow-up [11]. Recent evidence shows that CT provides diagnostic accuracy comparable to that of TEE in demonstrating vegetation > 10 mm in size and is more useful in detecting extravalvular complications such as abscesses [12].

Despite failure to grow an organism, it is possible that the vegetation was not completely sterilized in our patient. A penicillin-susceptible *Streptococcus* sp., such as in our patient, has a cure rate of > 95% [11]. It has been shown that vegetation size is predictive of response to medical treatment alone [13]. Robbins *et al.* observed that although 100% of vegetations under 10 mm responded to medical therapy alone, only 63% of vegetations over 10 mm did, with the rest requiring surgery. They postulated that as bacterial colonies deepen, they metabolize and proliferate slower, making certain antibiotics less efficacious [13]. This would explain the indolent, insidious course our patient experienced; his vegetation initially measured 30 × 25 mm. Furthermore, with increased size comes an increased risk of embolization [9, 14]. Despite treatment, vegetations over 10 mm embolize in 14% of cases versus 1% in vegetations under 10 mm [9].

Our patient’s indications for surgery were recurrent embolization, valve destruction, and large vegetation size. Other indications include persistent bacteremia despite antimicrobial therapy and abscess formation [8, 11].

When required, surgical options include debridement of the infected area with vegetation excision; valve repair; or, where unavoidable, valve replacement with a bioprosthetic valve [15]. Following surgery, outcomes are generally favorable, with two of the largest case series reporting that none of the nine cases described developing repeat vegetations after operative management [16, 17].

## Conclusions

In summary, we report a case of a patient with penicillin-susceptible *Streptococcus mitis* PV IE with a delayed diagnosis until echocardiography was considered, and which, despite appropriate antibiotic therapy, progressed several months later to cause recurrent pulmonary abscess and emboli leading to bioprosthetic valve replacement. This case report highlights the importance of echocardiography to diagnose causes of respiratory symptoms, the limitations in relying solely on inflammatory markers and echocardiography to survey treatment response, and the benefits of chest CT to monitor for systemic signs of right-sided IE relapse.
